# Erythropoietin in the General Population: Reference Ranges and Clinical, Biochemical and Genetic Correlates

**DOI:** 10.1371/journal.pone.0125215

**Published:** 2015-04-27

**Authors:** Niels Grote Beverborg, Niek Verweij, IJsbrand T. Klip, Haye H. van der Wal, Adriaan A. Voors, Dirk J. van Veldhuisen, Ron T. Gansevoort, Stephan J. L. Bakker, Pim van der Harst, Peter van der Meer

**Affiliations:** 1 Department of Cardiology, University Medical Center Groningen, University of Groningen, Groningen, The Netherlands; 2 Department of Nephrology, University Medical Center Groningen, University of Groningen, Groningen, The Netherlands; University of Florida, UNITED STATES

## Abstract

**Background:**

Although erythropoietin has been used for decades in the treatment of anemia, data regarding endogenous levels in the general population are scarce. Therefore, we determined erythropoietin reference ranges and its clinical, biochemical and genetic associations in the general population.

**Methods:**

We used data from 6,777 subjects enrolled in the Prevention of REnal and Vascular ENd-stage Disease (PREVEND) study. Fasting venous blood samples were obtained in the morning from all participants from 2001–2003. Serum erythropoietin concentrations were measured using a fully automated chemiluminescent enzyme-labeled immunometric assay. A genome-wide association study was performed to identify genetic determinants.

**Results:**

Mean age (± SD) was 53 ± 12 years and 50% were female. Median (IQR) erythropoietin concentrations were 7.6 (5.8–9.9) IU/L in men and 7.9 (6.0–10.6) IU/L in women. A strong positive correlation was found between erythropoietin and waist circumference, glucose and systolic blood pressure (all P < 0.05). In subjects with normal renal function there was a strong exponential relation between hemoglobin and erythropoietin, whereas in renal impairment (eGFR < 60 mL/min/1.73m^²^) this relation was linear (men) or absent (women) (P < 0.001 for interaction). Single-nucleotide polymorphisms at the *HBS1L-MYB* locus were shown to be related to erythropoietin levels (P < 9x10^-21^), more significantly than other erythrocyte parameters.

**Conclusion:**

We provide age-specific reference ranges for endogenous serum erythropoietin. Erythropoietin levels are positively associated with the components of the metabolic syndrome, except cholesterol. We show that even mild renal failure blunts erythropoietin production and propose the *HBS1L-MYB* locus as a regulator of erythropoietin.

## Introduction

Erythropoietin (EPO) is one of the primary regulators of erythropoiesis.[1,2] In the bone marrow, EPO promotes the proliferation of erythroid progenitor cells and increases the production of red blood cells.[2] While erythropoiesis normally proceeds at a low basal rate, EPO is capable of enhancing production as much as eightfold compared to the baseline rate. Eighty percent of EPO is produced in the kidney in reaction to impaired oxygen delivery, whereas the remainder is produced in the liver.[3] Various mechanisms decrease oxygen delivery to the kidney, including anemia, hypoperfusion due to arteriosclerosis or heart failure (HF) and decreased oxygen saturation due to several lung and cardiac diseases.[3]

Recombinant human EPO is intensively studied as therapeutic agent for anemia in oncology, renal disease and HF. In renal failure, the use of recombinant human EPO is indicated to correct anemia.[4] In HF patients however, a large trial conducted recently was not able to show beneficial effects on clinical outcome.[5] Administration of recombinant human EPO might even be hazardous in these patients as it increased the risk of thromboembolic events.[5]

The endogenous form of EPO has been studied in relatively small cohorts for its value as prognostic marker in chronic HF patients and the very elderly.[6,7] Data regarding endogenous EPO levels and its correlations with biochemical and genetic determinants are scarce. Assumptions are made based on studies in small, selected, often diseased populations.[8,9] For example, inflammation and aging are suggested to raise EPO levels[10,11], whereas diabetes lowers EPO levels.[12] Molecular regulation of EPO in hypoxia, by means of the hypoxia-inducible factors, has been well studied. Still, genetic associations of EPO levels in normoxic conditions are largely unexplored.

To gain understanding of the physiology of endogenous EPO levels, we studied its correlation with clinical, biochemical and genetic parameters in the Prevention of REnal and Vascular ENd-stage Disease (PREVEND) study, a large prospective, well characterized, observational cohort study. In addition, we provide age-specific reference ranges of serum EPO.

## Methods

### Study population

Participants from the PREVEND study were used for this study. Details of the protocol are described elsewhere.[13,14] In brief, PREVEND was designed to prospectively investigate the natural course of urinary albumin excretion (UAE) and its relationship with renal and cardiovascular disease in a large cohort drawn from the general population. From 1997 to 1998, all inhabitants of the city of Groningen (located at sea level), The Netherlands, aged 28–75 years (n = 85,421), were sent a questionnaire and a vial to collect an early morning urinary sample. In total, 40,856 subjects responded (47.8%). Subjects with type 1 diabetes mellitus (defined as the use of insulin) and pregnant women were excluded. The baseline PREVEND cohort (n = 8,592) was formed by subjects with an UAE ≥10 mg/L (n = 6,000) and a randomly selected control group with an UAE < 10 mg/L (n = 2,592). For the current analyses, we used data from the second survey between 2001 and 2003 (n = 6,984). We excluded 207 subjects because of missing EPO values.

A subset of the study population was selected to calculate reference ranges. Exclusion criteria used for the subset were: high-sensitive C-reactive protein (hs-CRP) ≥5 mg/L, estimated glomerular filtration rate (eGFR) < 60 mL/min/1.73m^2^, presence of anemia and/or heart failure, body-mass index (BMI) ≥30 kg/m^2^ and/or UAE > 10mg/L at the second survey, and/or the presence of liver disease, COPD and/or asthma at the second survey or in the preceding year. These criteria were used because EPO production might be impaired in renal and liver disease.[3,15] On the other hand, subjects with anemia, heart failure, lung disease, a hs-CRP ≥5 mg/L and/or a BMI ≥30 kg/m^2^ are expected to have higher than normal EPO values.[3,10,16,17]

For the genome wide association (GWA) study, we used 2,691 randomly selected non-anemic individuals. We excluded anemic individuals to avoid confounding variables. The PREVEND study has been approved by the medical ethics committee of the University Medical Center Groningen and was conducted in accordance with the Declaration of Helsinki. Written informed consent was obtained from all participants.

### Definitions

Anemia was defined according to the World Health Organization criteria as a hemoglobin level < 13.0 g/dL in men and < 12.0 g/dL in women. The glomerular filtration rate was estimated using the Chronic Kidney Disease Epidemiology Collaboration (CKD-EPI) formula based on serum creatinine levels.[18,19] Type 2 diabetes mellitus was defined as the use of anti-diabetic drugs, a fasting glucose level ≥7.0 mmol/L (126 mg/dL) or a non-fasting glucose level ≥11.1 mmol/L (200 mg/dL). Systolic and diastolic blood pressures were calculated as the mean of the last two out of ten measurements of the two visits. Hypertension was considered present when a subject had a systolic blood pressure ≥140 mmHg, a diastolic blood pressure ≥90 mmHg or when he or she was taking antihypertensive medication. BMI was calculated as the ratio of weight and height squared (kg/m2). Urinary albumin excretion was calculated as the average of two consecutive 24-h urine collections. All participants were asked about their current and former smoking habits. Current smoking or stopped smoking within the previous year was defined as smoking. A history of myocardial infarction and/or cerebrovascular accident was considered present if a subject reported hospital admission because of these conditions. Asthma, COPD, or liver disease were considered present if a subject received treatment for the condition or when the subject reported of having the disease. Standard 12-lead electrocardiograms were recorded using the computer Modular ECG Analysis System[20] and left ventricular hypertrophy (LVH) was defined using the Cornell criteria: RaVL + SV_3_ (with 6mm added in women) multiplied by the QRS duration.[21] LVH was defined as a value of > 2440 mm*ms.

### Analytical methods

Fasting blood samples were obtained in the morning from all participants from 2001–2003. We used serum stored at -80°C which was never thawed before assaying. Serum EPO levels were measured using the IMMULITE EPO assay (DPC, Los Angeles, California, USA)[22]. The assay consists of a ligandlabeled monoclonal anti-EPO capture antibody, an alkaline phosphatase-labeled polyclonal conjugate antibody, and solid-phase anti-ligand—coated polystyrene beads. A luminometer measured the amount of serum EPO using chemiluminescence. The assay showed an intra-assay variability of 2.3–5.0%, an inter-assay variability of 4.1–9.5% and a lower detection limit of 0.60 IU/L. Hemoglobin levels were measured using a Coulter Counter STKS sum (Coulter Corporation, Miami, Florida, USA). Concentrations of total cholesterol, plasma glucose and serum creatinine were measured using standard methods. Nephelometry was used to determine urinary albumin concentration and high-sensitive C-reactive protein (hs-CRP) (BN II, Dade Behring Diagnostica, Marburg, Germany). For urinary albumin concentration there was a threshold of 2.3 mg/L and an intra- and interassay variability of 2.2 and 2.6%, respectively. Hs-CRP had a lower detection limit of 0.175 mg/L and an intra- and interassay variability of less than 4.4 and 5.7%, respectively.

### Genome wide association study

Genotyping for PREVEND was performed on the Illumina CytoSNP12 v2 chip, a whole-genome scanning panel that includes up to 220,000 common genetic variants. Samples were excluded based on call rates below 0.95, gender mismatch, duplicate discordance and genetic similarity. Population stratification was assessed by principal component analysis over the sample correlation matrix, based on 16,842 independent, linkage disequilibrium-pruned single-nucleotide polymorphisms (SNP). Samples were excluded when they diverged from the mean with at least 3 standard deviations (Z-score > 3) for the first five principal components. SNPs were excluded with a minor allele frequency of <0.01, call rate <0.95, or deviation from Hardy Weinberg equilibrium (P < 1×10^-5^). The genome positions from the genotypes were converted from hg18 to hg19 using the UCSC LiftOver tool. Genome-wide genotype imputation was performed with SHAPEIT (v2 r644) and IMPUTE2 (v2.3.0) using 1,000 Genomes haplotypes Phase I integrated variant set release (v3, march 2012) in NCBI build 37 (hg19) as reference panel. Only imputed SNPs were used for the GWA.

### Statistical analyses

Data are presented as mean ± standard deviation when normally distributed, as median and interquartile range when non-normally distributed, and as frequencies and percentages for categorical variables. Baseline trends in EPO quintiles are evaluated using the extended Wilcoxon rank-sum test.[23] For further analyses, skewed variables were transformed to a logarithmic scale to achieve a normal distribution. References ranges in the subset were constructed using the median and 95% confidence interval (P2.5 and P97.5), stratified by 10-year age groups.

Univariable and multivariable regression were performed to assess clinical and biochemical associations with EPO. To be able to apply linear regression models, linearity is assessed using fractional polynomials. Consequently, hemoglobin is transformed as follows: (hemoglobin-14.5)^2. Univariable regression is adjusted for age, and associations are studied for men and women separately. All clinically important variables are included in the multivariable regression analysis. Interactions between hemoglobin, diabetes, hs-CRP, eGFR and UAE on EPO levels are analyzed because interactions between hemoglobin levels and diabetes, kidney and inflammation parameters are of possible clinical significance. Multivariable fractional polynomial regression is used to analyse the association between hemoglobin and EPO stratified for renal function and sex.

The GWA was performed on residuals of EPO levels after adjustment for age and gender using an additive genetic model in SNPTEST. We only considered SNPs with a minor allele frequency higher than 0.01.

We considered a p-value < 0.05 statistically significant for all analyses except the GWA. To adjust for multiple hypotheses testing, significance for the GWA was declared at P < 5 x 10^-8^. All models and analyses were performed using STATA version 12.0 (StataCorp LP, College Station, Texas, USA) and SNPTEST version 2.4.1.

## Results

### Characteristics of the study population

The baseline characteristics of the 6,777 patients, divided by EPO quintiles are presented in Table 1. Because of a significant interaction between hemoglobin and sex on EPO levels (p = 0.023), we additionally stratified baseline characteristics for sex, results are displayed in S1 and S2 Tables. Mean (± SD) age was 53.3 ± 12.1 years and approximately half (49.9%) of the patients were women. Median (IQR) EPO levels were 7.6 IU/L (5.8–9.9) in men and 7.9 IU/L (6.0–10.6) in women, mean hemoglobin concentrations were 14.4 ± 1.0 g/dL and 13.0 ± 1.0 g/dL, respectively. Subjects in the upper EPO quintiles were older, had a larger waist circumference, higher blood pressure and were less often smokers. Furthermore, a history of myocardial infarction or diabetes mellitus was more frequently observed with increasing EPO quintiles. Worse renal function and lower hemoglobin levels were observed in subjects with higher EPO levels; 13.1% of men and 33.9% of women in the highest EPO quintile were anemic. A total of 2,506 subjects passed the selection criteria for the reference set (see S3 Table for baseline characteristics of these subjects).

**Table 1:** Baseline characteristics.

Characteristic		Quintiles of erythropoietin					P-value for trend
	Total	1	2	3	4	5	
Erythropoietin, min—max	0.6–750.0	0.6–5.4	5.4–7.0	7.0–8.6	8.6–11.0	11.1–750.0	
*n*	6,777	1,363	1,351	1,366	1,352	1,345	
Erythropoietin (IU/L)	7.8 (5.9–10.3)	4.5 (3.7–5.0)	6.3 (5.9–6.6)	7.8 (7.4–8.2)	9.6 (9.1–10.3)	13.7 (12.2–16.7)	
**Demography**							
Age (years)	53.3 ± 12.1	51.7 ± 11.6	52.2 ± 11.8	52.9 ± 11.9	54.7 ± 12.4	54.9 ± 12.4	<0.001
Males (%)	50.1	53.4	53.2	49.8	50.9	43.1	<0.001
Waist circumference (cm)	92.2 ± 12.8	90.8 ± 11.7	91.3 ± 12.3	91.1 ± 12.5	93.7 ± 12.9	94.3 ± 14.0	<0.001
Systolic blood pressure (mmHg)	126.4 ± 18.8	125.3 ± 18.0	125.1 ± 18.3	125.2 ± 18.5	128.0 ± 19.3	128.5 ± 19.5	<0.001
Heart rate (bpm)	68.4 ± 10.0	69.0 ± 9.9	68.1 ± 9.8	68.1 ± 10.2	68.1 ± 9.9	68.9 ± 10.3	0.673
LVH according to Cornell (%)	2.1	1.6	1.8	2.4	2.5	2.4	0.068
**Baseline medical history**							
Smoking or quit <1 year (%)	30.3	35.8	32.2	30.8	28.1	24.7	<0.001
Myocardial infarction (%)	3.4	2.4	3.0	3.0	4.1	4.5	0.001
Stroke (%)	0.9	1.0	0.9	0.7	0.8	1.3	0.404
Venous thromboembolism (%)	0.6	0.3	0.7	0.5	1.1	0.6	0.124
Diabetes mellitus (%)	8.2	6.1	6.5	7.2	8.4	12.7	<0.001
**Laboratory values**							
Glucose (mmol/L)	5.1 ± 1.2	5.0 ± 0.9	5.0 ± 1.0	5.0 ± 1.2	5.1 ± 1.3	5.3 ± 1.5	<0.001
Cholesterol (mmol/L)	5.4 ± 1.0	5.5 ± 1.1	5.5 ± 1.1	5.5 ± 1.1	5.3 ± 1.0	5.3 ± 1.0	<0.001
eGFR (mL/min/1.73m^2^)	89.6 ± 17.8	91.1 ± 16.9	90.8 ± 17.0	90.2 ± 17.1	88.3 ± 18.3	87.5 ± 19.2	<0.001
UAE (mg/24h)	8.7 (6.1–15.7)	8.7 (6.2–14.8)	8.1 (6.0–14.1)	8.4 (6.0–14.8)	9.1 (6.1–17.3)	9.4 (6.3–19.0)	<0.001
hs-C-reactive protein (mg/L)	1.4 (0.6–3.0)	1.2 (0.6–2.6)	1.3 (0.6–2.8)	1.3 (0.6–2.8)	1.5 (0.7–3.3)	1.6 (0.7–3.8)	<0.001
Hemoglobin (g/dL)	13.7 ± 1.2	14.1 ± 1.1	13.9 ± 1.1	13.8 ± 1.1	13.7 ± 1.1	13.2 ± 1.4	<0.001
Anemia (%)	9.4	3.2	5.1	6.6	8.2	23.8	<0.001

LVH = Left Ventricular Hypertrophy, eGFR = estimated Glomerular Filtration Rate, UAE = Urinary Albumin Excretion

Values are given as means ± SD, medians (Q25–Q75) or proportions (%)

### References ranges for serum erythropoietin concentration by age

References ranges for serum EPO levels are displayed in Table 2. Substantial variation between subjects is reflected in the wide reference ranges. Serum EPO concentration showed a trend upwards over age (β = 0.005; 95% CI, 0.002–0.007)

**Table 2:** Reference ranges for serum erythropoietin (IU/L) levels per age group for subjects in the reference sample.

	Total (n = 2,506)		
Age, y	N (%)	Median	95% reference ranges (P2.5–P97.5)
<40	602 (24.0)	6.9	3.1–16.5
40–50	839 (33.5)	7.2	3.1–14.7
50–60	639 (25.5)	7.3	3.3–15.9
60–70	309 (12.3)	7.6	2.8–17.1
>70	117 (4.7)	7.5	4.0–17.9

### Clinical and biochemical associations

We performed univariable (S4 Table) and multivariable regression (Table 3) separate for men and women because of the significant interaction between hemoglobin and sex on EPO levels. In multivariable regression, the associations between EPO and hemoglobin, waist circumference, glucose and cholesterol were highly significant in both sexes. In men, age and a history of VTEs were independently associated with EPO. Additionally smoking was independently associated with lower EPO levels. Except for hemoglobin, no interactions with sex were observed. We analyzed interactions between hemoglobin and diabetes, UAE, eGFR and hs-CRP for the association with EPO. Multivariably, the interaction of hs-CRP and eGFR with hemoglobin in women and the interaction of hemoglobin with UAE in men were significant, other interactions were not. (S5 Table).

**Table 3:** Multivariate linear regression for erythropoietin.

	Men (n = 3,395)			Women (n = 3,382)			Interaction P-value
Variable	β*	95% Confidence interval	P-value	β*	95% Confidence interval	P-value	
Age (per 10 years)	0.048	0.022 to 0.074	<0.001	-0.008	-0.050 to 0.031	0.692	0.132
Waist circumference (cm)	0.006	0.003 to 0.008	<0.001	0.007	0.005 to 0.010	<0.001	0.294
Premenopausal	-	-	-	0.010	-0.069 to 0.088	0.813	-
UAE (mg/24h)†	0.010	-0.008 to 0.028	0.262	0.016	-0.007 to 0.040	0.179	0.328
Hs-C-reactive protein (mg/L)†	0.008	-0.009 to 0.024	0.371	0.010	-0.006 to 0.026	0.236	0.419
eGFR (per 10mL/min/1.73m^2^)	0.009	-0.008 to 0.025	0.319	-0.005	-0.021 to 0.012	0.587	0.713
[Hemoglobin (g/dL)– 14.5]^2^	0.063	0.053 to 0.074	<0.001	0.079	-0.072 to 0.085	<0.001	0.023
Glucose (mmol/L)	0.038	0.017 to 0.058	<0.001	0.029	0.005 to 0.053	0.017	0.866
Cholesterol (mmol/L)	-0.055	-0.078 to -0.032	<0.001	-0.041	-0.066 to -0.016	0.001	0.608
Smoking or quit <1 year	-0.078	-0.131 to -0.024	0.004	-0.058	-0.112 to -0.003	0.038	0.529
Systolic blood pressure (per 5mmHg)	0.001	-0.007 to 0.009	0.893	0.014	0.006 to 0.022	0.001	0.058
Heart rate (per 5b.p.m.)	-0.008	-0.020 to 0.004	0.168	-0.005	-0.018 to 0.008	0.454	0.367
Myocardial infarction	0.055	-0.050 to 0.160	0.303	0.114	-0.089 to 0.317	0.271	0.756
Stroke	0.063	-0.193 to 0.320	0.628	-0.107	-0.384 to 0.171	0.451	0.414
Venous thromboembolism	0.358	0.066 to 0.650	0.016	-0.041	-0.381 to 0.298	0.811	0.085

* The dependent variable erythropoietin was double log-transformed before included in the model. Therefore, a β of 1 should be interpreted as a doubling for each unit rise of the independent variable.

^†^ Both the dependent and independent variables were double log-transformed before included in the model. Therefore, a β of 1 should be interpreted as a doubling for each doubling of the independent variable.

UAE = Urinary Albumin Excretion, eGFR = estimated Glomerular Filtration Rate.

### Erythropoietin response in relation to kidney function

**Fig 1:**
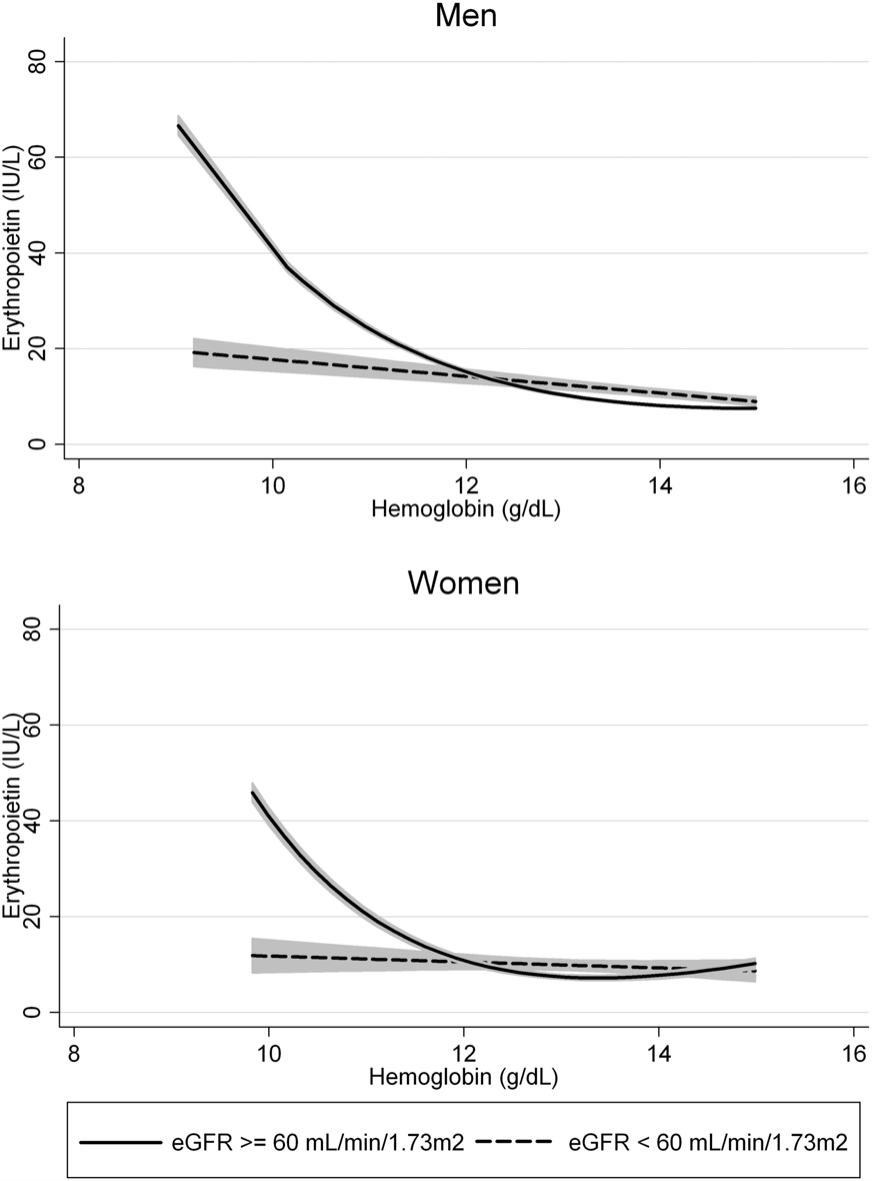
Relation between hemoglobin and erythropoietin in subjects with a normal or impaired renal function. Relation between hemoglobin and erythropoietin in men and women separated for normal (continuous line) and impaired renal function (dashed line) (eGFR ≥ or < 60 ml/min/1.73m^2^), the 95% confidence interval is depicted in grey.

In healthy subjects, EPO is almost entirely produced in the kidney. Therefore, we analyzed the effect of renal function on EPO levels. Three-way interaction between hemoglobin, the main driver of EPO production, renal function and sex on EPO was significant (P < 0.001). Additionally, we performed a fractional polynomial regression on hemoglobin and EPO in subjects with normal and impaired renal function (eGFR ≥ 60 or < 60 mL/min/1.73m^2^) separate for women and men. Results are shown in Fig 1. In both men and women, the relation was best described by a function with a power of -2, where the association of EPO with hemoglobin becomes steeper with lower hemoglobin levels. However, when renal function is impaired, only a linear increase in EPO was found in men, whereas the relation between EPO and hemoglobin was lost in women with impaired renal function (P = 0.250).

### Genetics determinants

**Fig 2:**
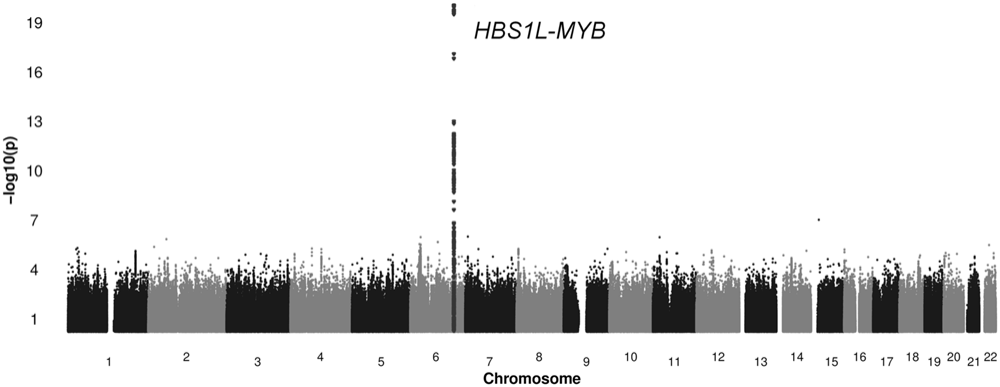
Association of serum erythropoietin with single-nucleotide polymorphisms (SNP). The -10log(P-value) is displayed on the y-axis, a higher value corresponds to a stronger association. Chromosome location is displayed on the x-axis. Each dot represents an individual SNP, the dark grey dots are the SNPs located in locus 6q23.

We performed a GWA study with 3,477,282 imputed autosomal SNPs (1000 genomes v3 reference panel) in 2,691 individuals on EPO levels. All genome wide significant SNPs were well imputed as shown in S6 Table. There was no evidence for inflation of test statistics at the final results (λ = 1.0156). The GWA identified a total of 108 SNPs significantly associated (P < 5.0x10^-8^) with EPO levels in one locus (6q23) on chromosome 6, as seen in Fig 2. We compared the association of rs7776054 with EPO and 6 other red blood cell traits. The minor allele of rs7776054 had the lowest P value for the association with higher EPO levels among red blood cell parameters [0.29 (95%CI 0.23 to 0.35) IU/L, P = 8.8x10-21]; mean corpuscular hemoglobin (P = 2.9x10^-10^), mean corpuscular volume (P = 1.6x10^-08^), packed cell volume (P = 2.9x10^-06^), mean corpuscular hemoglobin concentration (P = 0.001) and hemoglobin (P = 0.0003). The SNP rs7776054 could explain 3.1% of the total phenotypic variation of EPO levels, as reflected by the R^2^ of 0.031. The sentinel SNP is located near the *HBS1L* gene and is associated with the expression of 26 genes in trans (trans-eQTLs) at FDR < 0.05 but no genes in cis, as measured in non-transformed peripheral blood samples from 5,311 individuals (S7 Table).[24,25]

## Discussion

In the present study, we provide age-specific reference ranges for serum EPO in a large reference population. In this population, we observed EPO levels to increase over age. Furthermore, we showed that the association of hemoglobin with EPO levels in healthy subjects, especially in anemic conditions, is strongly dependent on renal function. Finally, we found a novel intergenic region that possibly regulates EPO production.

### Reference ranges

Since prior studies and the present one have shown an impaired EPO response to decreasing hemoglobin levels in renal disease [15], we excluded subjects with an impaired renal function from our reference subset. Thereby, subjects with other characteristics that could significantly influence EPO levels and would therefore not reflect the levels of healthy subjects were not included. Subjects with liver disease are excluded since 20% of EPO production is located in the liver.[3] We ruled out possible higher than normal EPO levels by excluding patients with anemia, COPD, asthma, heart failure, a hs-CRP ≥ 5 mg/L and/or a BMI ≥ 30 kg/m^2^.[3,10,11,16,17] The reference ranges, provided in the present study, are lower compared to reference ranges measured by others in small groups of healthy subjects.[9,26,27] Beguin et al. found a mean EPO level of 14.1 IU/L in 31 healthy subjects[26], a reference range of 2.21–20.95 IU/L was found in 200 healthy Thai adults[9] and 50 healthy Argentinean subjects had a EPO range of 5.3–34.0 IU/L, with a mean of 14 IU/L.[27] This difference may partially be explained by our selection of subjects. Also, we measured EPO in the morning in the fasting state, when EPO is at its circadian nadir [28]; no time of blood sampling was mentioned in the other studies. In another population of 85-year-old subjects, EPO levels were also higher.[7] However, 19.9% of the population was found to be anemic, which will notably increase the median EPO concentration, while we excluded these patients for the reference cohort. Additionally, the subjects were considerably older and erythropoietin levels are known to increase over age.[8] Indeed, in the reference cohort we observed that age was significantly correlated with EPO for both sexes. This observation is confirmed by a previous study from Ershler and colleagues in 143 healthy subjects with over time measurements.[8] They suggested the rise in EPO to be a compensation for subclinical blood loss, increased red blood cell turnover or increased EPO resistance of red cell precursors in older subjects. These high EPO levels might be the first sign of a subclinical disease or an “anemia of aging”.

### Biochemical correlates

The components of the metabolic syndrome, waist circumference, glucose and blood pressure are related to high EPO levels. Higher EPO levels in patients with the metabolic syndrome were seen before, and it was suggested that these high levels were caused by adipose tissue hypoxemia as EPO was related to obesity alone and not with glucose, blood pressure or lipid profile.[29] Interestingly, in our subjects, EPO is positively related with glucose and blood pressure in women as well. Therefore, EPO might not be related to the metabolic syndrome solely by hypoxia, but maybe by extra-hematopoietic pathways too. One suggestion is that EPO is up-regulated to protect pancreatic β-cells in high glucose conditions by exerting anti-apoptotic, proliferative, anti-inflammatory and angiogenic effects.[30] However, data regarding these functional EPO-receptors outside of the bone-marrow are highly controversial.[31]

In contrast to the other components of the metabolic syndrome, which are positively related to EPO, cholesterol levels are negatively associated. This might be because of the increased erythropoiesis in high EPO conditions, requiring more cholesterol. This is also seen in a study in renal transplant recipients and when administrating exogenous EPO, which improves the whole lipid profile.[32,33]

Between sexes, no significant differences in associations with EPO were found, except for the association of hemoglobin with EPO. In women, hemoglobin levels are generally lower while EPO levels are comparable to those in men. The association curve between hemoglobin and EPO has a similar shape, but is placed in a different hemoglobin range: compared to men, the association curve is shifted to the left in women, see S1 Fig In other words: a ‘normal’ EPO level of approximately 7 IU/L corresponds to a hemoglobin level of 15 g/dL in men, and 13.5 g/dL in women. The lower hemoglobin levels could be explained by menstrual blood loss. However, in that case, EPO levels would increase in response. The absence of such an increase in women at hemoglobin levels under the lower limit of men (14.0 g/dL) indicates that women have a lower set point for hemoglobin levels. Murphy et al. made the same observation and suggested that women could have a more efficient delivery of red cells to the capillary circulation, which explains the lower requirement for hemoglobin.[34]

Ershler et al. found the rise in EPO levels over age to be impaired in patients with a compromised renal function (e.g. due to diabetes mellitus or hypertension), which is thought to result in anemia.[8] We found an exponential increase of EPO levels with lowering hemoglobin levels in both men and women. However, this increase was linear and less strong in men with a reduced renal function and even absent in women with a reduced renal function. It is important to notice that, compared to subjects with a normal renal function, EPO levels are not lower in subjects with a low eGFR, but inappropriately low for the degree of anemia.[35] Even in these subjects with only a mildly impaired renal function, we found that EPO production is not able to rise sufficiently with lowering hemoglobin levels.

### Venous thromboembolic events

Elevated EPO levels caused by administration of exogenous EPO are known to be related to an increased risk of VTEs.[36] Recently, the RED-HF trial demonstrated more VTEs in HF patients treated with darbepoetin-α, a recombinant form of EPO.[5] A similar trial with darbepoetin-α in patients with diabetes, chronic kidney disease and anemia, the TREAT trial, found more strokes in the darbepoetin treated group, whereas a trend of more strokes was seen in the RED-HF trial.[37] It is thought that the administration of exogenous EPO increases blood viscosity and causes a higher blood pressure, the latter by means of endothelial vasoconstriction.[38–41] Hereby, the risk of clot forming is increased and more thromboembolic events are seen. In our cohort, we found no significant association in the linear regression model between endogenous EPO levels and a history of VTE or stroke, except for a significant association with a history of VTE in men. Additional logistic regression showed an OR of 1.259 for VTE and 1.203 for stroke. However, due to the limited number of subjects with a history of such an event, the confidence intervals are wide and include 1.000. Therefore we cannot demonstrate an association with EPO levels.

### Genetic associations

Genome wide association studies have been shown a powerful and unbiased tool for identifying novel biological mechanisms and pathways. We detected SNPs in chromosome region 6q23 near *HBS1L*-*MYB* to be significantly related to EPO levels, a locus with pleiotropic effects on other hematological parameters and predictive for severity in β thalassemia and sickle cell disease.[25,42] The sentinel SNP rs7776054 is in full linkage disequilibrium (1000G, European population) with a 3-nucleotide deletion, rs66650371, which is the second highest associated SNP and associated with the binding of multiple erythropoiesis-related transcription factors, suggesting this is the functional variant.[25] The strength of the association of rs7776054 with EPO is much stronger than the association with other red blood cell traits for which the SNP has been reported, which may suggest that the locus’s effect may be driven by changes in EPO levels, considering the function of EPO. Further analysis on gene-expression identified that the lead-SNP is associated with 26 genes in trans, many of which can be directly connected to the hematological system or red blood cell development. Our analyses strengthen the findings for the *HBS1L-MYB* locus as a master-regulator of erythropoiesis. However, future functional studies are necessary to gain insight into the underlying molecular mechanisms of EPO regulation.

### Strengths and limitations

Strengths of this study are the large size of the prospective community-based cohort and detailed information on many covariates. Since none of the subjects used recombinant human EPO, this drug did not affect our results. Finally, time of blood sampling was in the morning, so the influence of circadian rhythm on EPO levels was minimized.

Subjects from the PREVEND study are predominantly Caucasian. Therefore, our results cannot be extrapolated to subjects from other ethnicities. Second, EPO was only measured at one time point. Therefore, potential day-to-day variations could not be corrected for. Third, the lack of oxygen saturation or arterial oxygen measurements is a limitation, which inhibited us from further exploring underlying physiologic mechanisms that could explain the relation between multiple variables and EPO. Fourth, questionnaires were used to assess smoking status and the history of myocardial infarction and cerebrovascular events which might have biased the results. Finally, the PREVEND cohort is enriched for increased UAE. However, compared with the Framingham cohort, UAE was not higher in PREVEND.[43]

## Conclusion

We identified higher endogenous EPO levels to be positively correlated with the components of the metabolic syndrome. In addition, we identified genetic variation at the *HBS1L*-*MYB* locus to influence EPO levels, more significantly than other red blood cell parameters, suggesting these effects may be driven by EPO. Our results also suggest that EPO production in healthy subjects is dependent on renal function, as subjects with a mildly impaired renal function already show a blunted EPO response with lowering hemoglobin levels. Finally, we provide age-specific reference ranges of serum EPO levels.

## Supporting Information

S1 Table Baseline characteristics men. Values are given as means ± SD, medians (Q25–Q75) or proportions (%). LVH = Left Ventricular Hypertrophy, eGFR = estimated Glomerular Filtration Rate, UAE = Urinary Albumin Excretion.
(DOCX)

S2 Table Baseline characteristics women. Values are given as means ± SD, medians (Q25–Q75) or proportions (%). LVH = Left Ventricular Hypertrophy, eGFR = estimated Glomerular Filtration Rate, UAE = Urinary Albumin Excretion.
(DOCX)

S3 Table Baseline characteristics reference subjects. Values are given as means ± SD, medians (Q25–Q75) or proportions (%). LVH = Left Ventricular Hypertrophy, eGFR = estimated Glomerular Filtration Rate, UAE = Urinary Albumin Excretion.
(DOCX)

S4 Table Univariable linear regression for erythropoietin adjusted for age. * The dependent variable erythropoietin was double log-transformed before included in the model. Therefore, a β of 1 should be interpreted as a doubling for each unit rise of the independent variable. † Both the dependent and independent variables were double log-transformed before included in the model. Therefore, a β of 1 should be interpreted as a doubling for each doubling of the independent variable. UAE = Urinary Albumin Excretion, eGFR = estimated Glomerular Filtration Rate.
(DOCX)

S5 Table Multivariable interaction on erythropoietin. * The dependent variable erythropoietin was double log-transformed before included in the model. Therefore, a β of 1 should be interpreted as a doubling for each unit rise of the independent variable. † Both the dependent and independent variables were double log-transformed before included in the model. Therefore, a β of 1 should be interpreted as a doubling for each doubling of the independent variable. § All clinically important variables are included in the regression model. UAE = Urinary Albumin Excretion, eGFR = estimated Glomerular Filtration Rate.
(DOCX)

S6 Table Genome wide significant SNPs for EPO levels. (DOCX)

S7 Table Significant Trans eQTL’s (FDR < 0.05) for rs7776054 detected in non-transformed peripheral blood samples from 5,311 individuals. (DOCX)

S1 Fig Association of hemoglobin and erythropoietin for men and women. The association between hemolgobin and erythropoietin separate for men (solid line) and women (dashed line); the shift of the association is depicted by the gray arrow. The normal range of hemoglobin levels is displayed by the black lines with both sided arrow heads.
(TIF)
