# Supplementary material for: Erythropoietin in the General Population: Reference Ranges and Clinical, Biochemical and Genetic Correlates
Source: PLoS One. 2015 Apr 27;10(4):e0125215. doi: 10.1371/journal.pone.0125215 (PMC4411129; doi:10.1371/journal.pone.0125215)
Supplement: S1 Table — Values are given as means ± SD, medians (Q25–Q75) or proportions (%). LVH = Left Ventricular Hypertrophy, eGFR = estimated Glomerular Filtration Rate, UAE = Urinary Albumin Excretion. (DOCX) [file pone.0125215.s001.docx]

| **Supplemental Data Table 1: Baseline characteristics men** | | | | | | | |
| --- | --- | --- | --- | --- | --- | --- | --- |
| **Characteristic** |  | **Quintiles of erythropoietin** | | | | | **P-value for trend** |
|  | **Total** | **1** | **2** | **3** | **4** | **5** |  |
| Erythropoietin, min – max |  | 0.6 – 5.3 | 5.3 – 6.8 | 6.8 - 8.3 | 8.4 – 10.6 | 10.7 - 205 |  |
| *n* | 3,395 | 684 | 679 | 679 | 680 | 673 |  |
| Erythropoietin (IU/L) | 7.6 (5.8 – 9.9) | 4.4 (3.7 – 4.9) | 6.2 (5.8 – 6.5) | 7.6 (7.2 – 8.0) | 9.3 (8.8 – 9.9) | 13.0 (11.6 – 15.4) |  |
| Demography |  |  |  |  |  |  |  |
| Age (years) | 54.2 ± 12.4 | 51.8 ± 11.9 | 52.8 ± 12.3 | 53.2 ± 11.9 | 55.7 ± 12.7 | 57.8 ± 12.4 | <0.001 |
| Waist circumference (cm) | 97.2 ± 11.1 | 95.1 ± 10.0 | 96.5 ± 10.9 | 96.2 ± 10.8 | 98.0 ± 11.3 | 100.1 ± 11.9 | <0.001 |
| Systolic blood pressure(mmHg) | 130.5 ± 17.6 | 129.1 ± 17.0 | 128.7 ± 17.0 | 129.6 ± 17.2 | 131.8 ± 18.0 | 133.6 ± 18.2 | <0.001 |
| Heart rate (bpm) | 67.2 ± 10.3 | 67.4 ± 10.0 | 67.4 ± 10.3 | 66.6 ± 9.9 | 67.1 ± 10.4 | 67.5 ± 11.1 | 0.567 |
| LVH according to Cornell (%) | 3.0 | 2.2 | 2.4 | 3.5 | 3.7 | 3.3 | 0.093 |
| Baseline medical history |  |  |  |  |  |  |  |
| Smoking or quit <1 year (%) | 30.1 | 34.8 | 31.2 | 30.8 | 28.8 | 25.0 | <0.001 |
| Myocardial infarction (%) | 5.5 | 3.5 | 4.6 | 5.3 | 6.5 | 7.4 | <0.001 |
| Stroke (%) | 1.0 | 0.9 | 0.7 | 1.2 | 0.9 | 1.5 | 0.268 |
| Venous thromboembolism (%) | 0.7 | 0.3 | 0.6 | 0.3 | 1.6 | 0.7 | 0.057 |
| Diabetes mellitus (%) | 9.3 | 8.0 | 6.5 | 8.5 | 9.2 | 14.3 | <0.001 |
| Laboratory values |  |  |  |  |  |  |  |
| Glucose (mmol/L) | 5.2 ± 1.2 | 5.1 ± 1.0 | 5.1 ± 1.1 | 5.1 ± 1.2 | 5.2 ± 1.2 | 5.4 ± 1.6 | <0.001 |
| Cholesterol (mmol/L) | 5.4 ± 1.0 | 5.5 ± 1.0 | 5.5 ± 1.0 | 5.5 ± 1.1 | 5.3 ± 1.0 | 5.3 ± 1.0 | <0.001 |
| eGFR (mL/min/1.73m²) | 88.3 ± 18.0 | 90.4 ± 17.0 | 90.5 ± 16.9 | 89.4 ± 16.9 | 86.8 ± 18.6 | 84.4 ± 19.5 | <0.001 |
| UAE (mg/24h) | 9.8 (6.7 – 20.8) | 9.4 (6.7 – 17.8) | 8.9 (6.3 – 17.2) | 9.3 (6.4 – 17.4) | 11.2 (6.9 – 22.4) | 11.6 (7.1 – 31.5) | <0.001 |
| hs-C-reactive protein (mg/L) | 1.3 (0.6 – 2.8) | 1.2 (0.6 – 2.5) | 1.1 (0.6 – 2.6) | 1.2 (0.6 – 2.5) | 1.3 (0.6 – 3.0) | 1.5 (0.7 – 3.5) | <0.001 |
| Hemoglobin (g/dL) | 14.4 ± 1.0 | 14.7 ± 0.9 | 14.5 ± 0.8 | 14.5 ± 0.9 | 14.4 ± 0.9 | 14.1 ± 1.2 | <0.001 |
| Anemia (%) | 5.4 | 2.5 | 2.8 | 3.8 | 5.0 | 13.1 | <0.001 |
| Values are given as means ± SD, medians (Q25 – Q75) or proportions (%)  LVH = Left Ventricular Hypertrophy, eGFR = estimated Glomerular Filtration Rate, UAE = Urinary Albumin Excretion | | | | | | | |
