# Supplementary material for: Erythropoietin in the General Population: Reference Ranges and Clinical, Biochemical and Genetic Correlates
Source: PLoS One. 2015 Apr 27;10(4):e0125215. doi: 10.1371/journal.pone.0125215 (PMC4411129; doi:10.1371/journal.pone.0125215)
Supplement: S2 Table — Values are given as means ± SD, medians (Q25–Q75) or proportions (%). LVH = Left Ventricular Hypertrophy, eGFR = estimated Glomerular Filtration Rate, UAE = Urinary Albumin Excretion. (DOCX) [file pone.0125215.s002.docx]

| **Supplemental Data Table 2: Baseline characteristics women** | | | | | | | |
| --- | --- | --- | --- | --- | --- | --- | --- |
| **Characteristic** |  | **Quintiles of erythropoietin** | | | | | **P-value for trend** |
|  | **Total** | **1** | **2** | **3** | **4** | **5** |  |
| Erythropoietin, min – max |  | 0.6 – 5.6 | 5.6 – 7.2 | 7.2 – 8.8 | 8.9 – 11.4 | 11.5 - 750 |  |
| *n* | 3,382 | 681 | 672 | 679 | 684 | 666 |  |
| Erythropoietin (IU/L) | 7.9 (6.0 – 10.6) | 4.6 (3.8 – 5.1) | 6.4 (6.0 – 6.8) | 7.9 (7.6 – 8.4) | 10.0 (9.4 – 10.7) | 14.5 (12.9 – 18.2) |  |
| Demography |  |  |  |  |  |  |  |
| Age (years) | 52.3 ± 11.6 | 51.5 ± 11.1 | 51.3 ± 11.3 | 52.7 ±`11.6 | 53.8 ± 12.1 | 52.2 ± 11.9 | 0.013 |
| Waist circumference (cm) | 87.3 ± 12.4 | 85.4 ± 11.1 | 85.5 ± 11.4 | 86.3 ± 12.1 | 89.0 ± 12.9 | 90.0 ± 13.7 | <0.001 |
| Systolic blood pressure (mmHg) | 122.3 ± 19.0 | 120.7 ± 17.9 | 120.7 ± 19.0 | 121.3 ± 18.6 | 123.9 ± 19.6 | 124.7 ± 19.6 | <0.001 |
| Heart rate (bpm) | 69.6 ± 9.5 | 70.6 ± 9.5 | 69.3 ± 9.2 | 69.6 ± 10.3 | 69.1 ± 8.9 | 69.7 ± 9.6 | 0.138 |
| LVH according to Cornell (%) | 1.3 | 1.0 | 0.9 | 1.3 | 1.0 | 2.1 | 0.098 |
| Baseline medical history |  |  |  |  |  |  |  |
| Smoking or quit <1 year (%) | 30.5 | 36.1 | 33.0 | 30.9 | 27.2 | 25.2 | <0.001 |
| Myocardial infarction (%) | 1.3 | 1.0 | 0.9 | 1.0 | 1.6 | 2.1 | 0.041 |
| Stroke (%) | 0.8 | 1.0 | 0.7 | 0.6 | 0.6 | 1.2 | 0.877 |
| Venous thromboembolism (%) | 0.6 | 0.3 | 0.9 | 0.3 | 0.9 | 0.4 | 0.732 |
| Diabetes mellitus (%) | 7.1 | 3.8 | 6.8 | 6.5 | 7.0 | 11.2 | <0.001 |
| Laboratory values |  |  |  |  |  |  |  |
| Glucose (mmol/L) | 4.9 ± 1.1 | 4.8 ± 0.9 | 4.8 ± 0.9 | 4.9 ± 1.2 | 5.0 ± 1.2 | 5.2 ± 1.4 | <0.001 |
| Cholesterol (mmol/L) | 5.4 ± 1.1 | 5.6 ± 1.1 | 5.5 ± 1.1 | 5.5 ± 1.1 | 5.4 ± 1.0 | 5.3 ± 1.0 | <0.001 |
| eGFR (mL/min/1.73m²) | 90.9 ± 17.5 | 91.8 ± 16.7 | 91.7 ± 17.2 | 91.0 ± 16.9 | 89.7 ± 17.8 | 88.3 ± 18.0 | 0.044 |
| UAE (mg/24h) | 7.8 (5.7 – 12.6) | 7.8 (5.8 – 11.2) | 7.6 (5.9 – 12.8) | 7.4 (5.6 – 11.9) | 7.9 (5.6 – 13.1) | 8.2 (5.8 -13.3) | 0.064 |
| hs-C-reactive protein (mg/L) | 1.4 (0.6 – 3.2) | 1.2 (0.5 – 2.7) | 1.4 (0.6 – 3.0) | 1.4 (0.6 – 3.2) | 1.5 (0.7 – 3.7) | 1.6 (0.7 – 4.0) | <0.001 |
| Hemoglobin (g/dL) | 13.0 ± 1.0 | 13.4 ± 0.9 | 13.2 ± 0.9 | 13.1 ± 0.9 | 13.0 ± 0.9 | 12.4 ± 1.2 | <0.001 |
| Anemia (%) | 13.3 | 3.9 | 8.1 | 10.1 | 11.1 | 33.9 | <0.001 |
| Values are given as means ± SD, medians (Q25 – Q75) or proportions (%)  LVH = Left Ventricular Hypertrophy, eGFR = estimated Glomerular Filtration Rate, UAE = Urinary Albumin Excretion | | | | | | | |
