# Supplementary material for: Erythropoietin in the General Population: Reference Ranges and Clinical, Biochemical and Genetic Correlates
Source: PLoS One. 2015 Apr 27;10(4):e0125215. doi: 10.1371/journal.pone.0125215 (PMC4411129; doi:10.1371/journal.pone.0125215)
Supplement: S3 Table — Values are given as means ± SD, medians (Q25–Q75) or proportions (%). LVH = Left Ventricular Hypertrophy, eGFR = estimated Glomerular Filtration Rate, UAE = Urinary Albumin Excretion. (DOCX) [file pone.0125215.s003.docx]

| **Supplemental Data Table 3: Baseline characteristics reference subjects** | |
| --- | --- |
| **Characteristic** | **Total (n = 2,506)** |
| Demography |  |
| Age (years) | 49.5 ± 10.9 |
| Males (%) | 49.7 |
| Waist circumference (cm) | 87.3 ± 9.8 |
| Systolic blood pressure (mmHg) | 120.3 ± 15.3 |
| Heart rate (bpm) | 67.8 ± 9.7 |
| LVH according to Cornell (%) | 1.8 |
| Baseline medical history |  |
| Smoking or quit <1 year (%) | 29.7 |
| Myocardial infarction (%) | 1.2 |
| Stroke (%) | 0.6 |
| Venous thromboembolism (%) | 0.4 |
| Diabetes mellitus (%) | 4.0 |
| Laboratory values |  |
| Glucose (mmol/L) | 4.8 ± 0.8 |
| Cholesterol (mmol/L) | 5.4 ± 1.0 |
| eGFR (mL/min/1.73m²) | 93.6 ± 14.8 |
| UAE (mg/24h) | 6.3 (5.3 – 7.8) |
| hs-C-reactive protein (mg/L) | 0.9 (0.4 – 1.6) |
| Erythropoietin (IU/L) | 7.2 (5.5 – 9.2) |
| Hemoglobin (g/dL) | 13.8 ± 1.0 |
| Values are given as means ± SD, medians (Q25 – Q75) or proportions (%).  LVH = Left Ventricular Hypertrophy, eGFR = estimated Glomerular Filtration Rate  UAE = Urinary Albumin Excretion | |
