# Supplementary material for: Erythropoietin in the General Population: Reference Ranges and Clinical, Biochemical and Genetic Correlates
Source: PLoS One. 2015 Apr 27;10(4):e0125215. doi: 10.1371/journal.pone.0125215 (PMC4411129; doi:10.1371/journal.pone.0125215)
Supplement: S4 Table — * The dependent variable erythropoietin was double log-transformed before included in the model. Therefore, a β of 1 should be interpreted as a doubling for each unit rise of the independent variable. † Both the dependent and independent variables were double log-transformed before included in the model. Therefore, a β of 1 should be interpreted as a doubling for each doubling of the independent variable. UAE = Urinary Albumin Excretion, eGFR = estimated Glomerular Filtration Rate. (DOCX) [file pone.0125215.s004.docx]

| **Supplemental Data Table 4. Univariable linear regression for erythropoietin adjusted for age** | | | | | | |
| --- | --- | --- | --- | --- | --- | --- |
|  |  | **Men (n= 3,395)** |  |  | **Women (n= 3,382)** |  |
| **Variable** | **β*** | **95% Confidence interval** | **P-value** | **β*** | **95% Confidence interval** | **P-value** |
| Waist circumference | 0.006 | 0.004 to 0.008 | <0.001 | 0.008 | 0.006 to 0.010 | <0.001 |
| Premenopausal | - | - | - | 0.133 | 0.052 to 0.213 | 0.001 |
| UAE ( mg/24h)† | 0.022 | 0.007 to 0.037 | 0.004 | 0.042 | 0.020 to 0.063 | <0.001 |
| Hs-C-reactive protein (mg/L)† | 0.015 | -0.001 to 0.030 | 0.066 | 0.027 | 0.011 to 0.043 | 0.001 |
| eGFR (per 10mL/min/1.73m²) | -0.020 | -0.036 to -0.005 | 0.011 | -0.003 | -0.020 to 0.014 | 0.723 |
| Anemia | 0.536 | 0.441 to 0.631 | <0.001 | 0.737 | 0.667 to 0.808 | <0.001 |
| [Hemoglobin (g/dL) – 14.5]² | 0.064 | 0.055 to 0.074 | <0.001 | 0.085 | 0.079 to 0.091 | <0.001 |
| Diabetes | 0.109 | 0.032 to 0.185 | 0.005 | 0.222 | 0.123 to 0.322 | <0.001 |
| Glucose (mmol/L) | 0.037 | 0.019 to 0.055 | <0.001 | 0.061 | 0.038 to 0.084 | <0.001 |
| Cholesterol (mmol/L) | -0.056 | -0.077 to -0.035 | <0.001 | -0.087 | -0.112 to -0.061 | <0.001 |
| Smoking or quit <1 year | -0.086 | -0.134 to -0.038 | <0.001 | -0.130 | -0.185 to -0.075 | <0.001 |
| Systolic blood pressure (per 5mmHg) | 0.005 | -0.002 to 0.011 | 0.184 | 0.015 | 0.007 to 0.022 | <0.001 |
| Heart rate (per 5b.p.m.) | 0.000 | -0.010 to 0.011 | 0.978 | -0.008 | -0.021 to 0.005 | 0.226 |
| Myocardial infarction | 0.105 | 0.007 to 0.203 | 0.035 | 0.215 | 0.005 to 0.435 | 0.056 |
| Stroke | 0.182 | -0.032 to 0.398 | 0.096 | 0.087 | -0.190 to 0.365 | 0.536 |
| Venous thromboembolism | 0.226 | -0.034 to 0.485 | 0.088 | 0.068 | -0.268 to 0.404 | 0.691 |
| * The dependent variable erythropoietin was double log-transformed before included in the model. Therefore, a β of 1 should be interpreted as a doubling for each unit rise of the independent variable.  † Both the dependent and independent variables were double log-transformed before included in the model. Therefore, a β of 1 should be interpreted as a doubling for each doubling of the independent variable.  UAE = Urinary Albumin Excretion, eGFR = estimated Glomerular Filtration Rate. | | | | | | |
