# Supplementary material for: Erythropoietin in the General Population: Reference Ranges and Clinical, Biochemical and Genetic Correlates
Source: PLoS One. 2015 Apr 27;10(4):e0125215. doi: 10.1371/journal.pone.0125215 (PMC4411129; doi:10.1371/journal.pone.0125215)
Supplement: S5 Table — * The dependent variable erythropoietin was double log-transformed before included in the model. Therefore, a β of 1 should be interpreted as a doubling for each unit rise of the independent variable. † Both the dependent and independent variables were double log-transformed before included in the model. Therefore, a β of 1 should be interpreted as a doubling for each doubling of the independent variable. § All clinically important variables are included in the regression model. UAE = Urinary Albumin Excretion, eGFR = estimated Glomerular Filtration Rate. (DOCX) [file pone.0125215.s005.docx]

| **Supplemental Data Table 5. Multivariable interaction on erythropoietin** | | | | | | |
| --- | --- | --- | --- | --- | --- | --- |
|  |  | **Men (n= 3,395)** |  |  | **Women (n= 3,382)** |  |
| **Interactions §** | **β*** | **95% Confidence interval** | **P-value** | **β*** | **95% Confidence interval** | **P-value** |
| [Hemoglobin (g/dL) – 14.5]² * Diabetes | 0.007 | -0.022 to 0.036 | 0.623 | 0.013 | -0.018 to 0.043 | 0.410 |
| [Hemoglobin (g/dL) – 14.5]² * Hs-C-reactive protein (mg/L)† | -0.005 | -0.010 to 0.000 | 0.053 | -0.008 | -0.011 to -0.005 | <0.001 |
| [Hemoglobin (g/dL) – 14.5]² * UAE ( mg/24h)† | -0.005 | -0.009 to -0.001 | 0.011 | 0.001 | -0.005 to 0.006 | 0.823 |
| [Hemoglobin (g/dL) – 14.5]² * eGFR (per 10mL/min/1.73m²) | 0.003 | -0.001 to 0.007 | 0.135 | 0.006 | 0.002 to 0.010 | 0.001 |
| UAE ( mg/24h)† * eGFR (per 10mL/min/1.73m²) | 0.002 | -0.005 to 0.008 | 0.591 | 0.002 | -0.007 to 0.011 | 0.691 |
| UAE ( mg/24h)† * Hs-C-reactive protein (mg/L)† | 0.002 | -0.008 to 0.012 | 0.762 | -0.012 | -0.026 to 0.001 | 0.067 |
| eGFR (per 10mL/min/1.73m²) * Hs-C-reactive protein (mg/L)† | -0.004 | -0.012 to 0.004 | 0.352 | 0.005 | -0.003 to 0.014 | 0.196 |
| * The dependent variable erythropoietin was double log-transformed before included in the model. Therefore, a β of 1 should be interpreted as a doubling for each unit rise of the independent variable.  † Both the dependent and independent variables were double log-transformed before included in the model. Therefore, a β of 1 should be interpreted as a doubling for each doubling of the independent variable.  **§** All clinically important variables are included in the regression model.  UAE = Urinary Albumin Excretion, eGFR = estimated Glomerular Filtration Rate. | | | | | | |
