# Supplementary material for: Erythropoietin in the General Population: Reference Ranges and Clinical, Biochemical and Genetic Correlates
Source: PLoS One. 2015 Apr 27;10(4):e0125215. doi: 10.1371/journal.pone.0125215 (PMC4411129; doi:10.1371/journal.pone.0125215)
Supplement: S6 Table — (DOCX) [file pone.0125215.s006.docx]

| **Supplemental Data Table 6: Genome wide significant SNPs for EPO levels** | | | | | | | | | | |
| --- | --- | --- | --- | --- | --- | --- | --- | --- | --- | --- |
| **SNP ID** | **Position**  **(chr6)** | **Non coding allele** | **Coding allele** | **INFO** | **N** | **Coded allele FRQ** | **HWE** | **P-value** | **Beta** | **SE** |
| rs7776054 | 135418916 | A | G | 0.99 | 2691 | 0.26 | 0.40 | 8.77E-21 | 0.29 | 0.03 |
| **chr6:135418632:D (**[rs66650371](http://www.broadinstitute.org/mammals/haploreg/detail_v2.php?query=&id=rs66650371)) | **135418632** | **TTAC** | **T** | **0.99** | **2691** | **0.26** | **0.40** | **8.95E-21** | **0.29** | **0.03** |
| rs9399137 | 135419018 | T | C | 0.99 | 2691 | 0.26 | 0.43 | 9.68E-21 | 0.29 | 0.03 |
| rs35786788 | 135419042 | G | A | 0.99 | 2691 | 0.26 | 0.43 | 9.69E-21 | 0.29 | 0.03 |
| rs9389268 | 135419631 | A | G | 0.99 | 2691 | 0.26 | 0.40 | 9.74E-21 | 0.29 | 0.03 |
| rs9376091 | 135419636 | C | T | 0.99 | 2691 | 0.26 | 0.40 | 9.76E-21 | 0.29 | 0.03 |
| rs9402685 | 135419688 | T | C | 0.99 | 2691 | 0.26 | 0.40 | 1.09E-20 | 0.29 | 0.03 |
| rs34164109 | 135421176 | C | T | 0.99 | 2691 | 0.26 | 0.37 | 1.18E-20 | 0.29 | 0.03 |
| rs9376090 | 135411228 | T | C | 1.00 | 2691 | 0.26 | 0.42 | 1.73E-20 | 0.29 | 0.03 |
| rs9402686 | 135427817 | G | A | 1.00 | 2691 | 0.27 | 0.38 | 1.76E-20 | 0.28 | 0.03 |
| rs11759553 | 135422296 | A | T | 0.99 | 2691 | 0.28 | 0.33 | 1.77E-20 | 0.28 | 0.03 |
| rs7758845 | 135428537 | A | C | 0.99 | 2691 | 0.27 | 0.38 | 1.93E-20 | 0.29 | 0.03 |
| rs9389269 | 135427159 | T | C | 1.00 | 2691 | 0.27 | 0.38 | 2.06E-20 | 0.28 | 0.03 |
| rs4895441 | 135426573 | A | G | 1.00 | 2691 | 0.27 | 0.36 | 2.13E-20 | 0.28 | 0.03 |
| rs9376092 | 135427144 | C | A | 1.00 | 2691 | 0.27 | 0.38 | 2.17E-20 | 0.28 | 0.03 |
| rs9373124 | 135423209 | T | C | 0.99 | 2691 | 0.28 | 0.33 | 2.20E-20 | 0.28 | 0.03 |
| rs35959442 | 135424179 | C | G | 1.00 | 2691 | 0.27 | 0.33 | 2.28E-20 | 0.28 | 0.03 |
| rs4895440 | 135426558 | A | T | 1.00 | 2691 | 0.27 | 0.33 | 2.43E-20 | 0.28 | 0.03 |
| rs1331309 | 135406178 | T | G | 0.98 | 2691 | 0.26 | 0.42 | 2.86E-20 | 0.29 | 0.03 |
| rs9399136 | 135402339 | T | C | 0.98 | 2691 | 0.26 | 0.48 | 3.13E-20 | 0.29 | 0.03 |
| rs6920211 | 135431318 | T | C | 0.97 | 2691 | 0.25 | 0.24 | 7.99E-18 | 0.28 | 0.03 |
| rs9494142 | 135431640 | T | C | 0.98 | 2691 | 0.24 | 0.17 | 1.52E-17 | 0.28 | 0.03 |
| rs9494145 | 135432552 | T | C | 1.00 | 2691 | 0.22 | 0.34 | 1.60E-16 | 0.27 | 0.03 |
| rs9483788 | 135435501 | T | C | 0.98 | 2691 | 0.24 | 0.52 | 9.92E-14 | 0.24 | 0.03 |
| rs6934903 | 135451564 | T | A | 0.96 | 2691 | 0.17 | 0.74 | 1.35E-13 | 0.27 | 0.04 |
| rs2223385 | 135435171 | G | A | 0.98 | 2691 | 0.23 | 0.47 | 5.65E-13 | -0.23 | 0.03 |
| rs1411919 | 135432061 | A | G | 0.99 | 2691 | 0.23 | 0.48 | 6.38E-13 | -0.23 | 0.03 |
| rs6569992 | 135452152 | G | A | 0.96 | 2691 | 0.18 | 0.65 | 6.60E-13 | 0.26 | 0.04 |
| rs6924609 | 135440657 | A | G | 0.98 | 2691 | 0.23 | 0.38 | 7.79E-13 | -0.23 | 0.03 |
| rs9483789 | 135441165 | T | C | 0.98 | 2691 | 0.23 | 0.38 | 7.89E-13 | -0.23 | 0.03 |
| rs10457632 | 135445202 | A | G | 0.97 | 2691 | 0.23 | 0.44 | 8.01E-13 | -0.23 | 0.03 |
| rs9389270 | 135431807 | C | T | 0.99 | 2691 | 0.23 | 0.48 | 8.55E-13 | -0.23 | 0.03 |
| rs10457631 | 135445179 | A | C | 0.97 | 2691 | 0.22 | 0.44 | 8.72E-13 | -0.23 | 0.03 |
| rs6925841 | 135441245 | C | T | 0.98 | 2691 | 0.23 | 0.38 | 8.90E-13 | -0.23 | 0.03 |
| rs1320959 | 135437386 | T | C | 0.99 | 2691 | 0.23 | 0.41 | 1.14E-12 | -0.23 | 0.03 |
| rs9389271 | 135433404 | C | T | 0.99 | 2691 | 0.23 | 0.51 | 1.22E-12 | -0.23 | 0.03 |
| rs9483791 | 135442525 | T | C | 0.98 | 2691 | 0.23 | 0.35 | 1.26E-12 | -0.23 | 0.03 |
| rs1320962 | 135443144 | G | A | 0.98 | 2691 | 0.23 | 0.35 | 1.34E-12 | -0.23 | 0.03 |
| rs1320963 | 135443212 | A | G | 0.98 | 2691 | 0.23 | 0.35 | 1.35E-12 | -0.23 | 0.03 |
| rs2026938 | 135444009 | G | A | 0.98 | 2691 | 0.23 | 0.35 | 1.61E-12 | -0.23 | 0.03 |
| rs9376093 | 135445448 | C | T | 0.98 | 2691 | 0.23 | 0.38 | 1.81E-12 | -0.23 | 0.03 |
| rs9494154 | 135468005 | A | T | 1.00 | 2691 | 0.17 | 0.89 | 1.87E-12 | 0.25 | 0.04 |
| rs9376094 | 135445814 | T | A | 0.98 | 2691 | 0.23 | 0.38 | 2.00E-12 | -0.23 | 0.03 |
| rs55875437 | 135467188 | C | T | 1.00 | 2691 | 0.17 | 0.95 | 2.13E-12 | 0.25 | 0.04 |
| rs75623964 | 135468310 | C | T | 1.00 | 2691 | 0.17 | 0.84 | 2.25E-12 | 0.25 | 0.04 |
| rs55654242 | 135477987 | T | A | 0.96 | 2691 | 0.17 | 0.69 | 2.30E-12 | 0.26 | 0.04 |
| rs6924687 | 135467521 | T | A | 1.00 | 2691 | 0.17 | 0.84 | 2.41E-12 | 0.25 | 0.04 |
| rs147519702 | 135474276 | A | G | 0.98 | 2691 | 0.17 | 0.69 | 2.60E-12 | 0.26 | 0.04 |
| rs17064262 | 135465474 | T | C | 1.00 | 2691 | 0.17 | 0.89 | 3.38E-12 | 0.25 | 0.04 |
| rs7738935 | 135473815 | C | T | 0.98 | 2691 | 0.17 | 0.68 | 3.41E-12 | 0.25 | 0.04 |
| rs7751525 | 135463708 | A | G | 0.99 | 2691 | 0.17 | 0.95 | 3.49E-12 | 0.25 | 0.04 |
| rs145425865 | 135476121 | A | C | 0.98 | 2691 | 0.17 | 0.63 | 3.71E-12 | 0.25 | 0.04 |
| rs11960991 | 135476551 | G | A | 0.98 | 2691 | 0.17 | 0.63 | 3.78E-12 | 0.25 | 0.04 |
| rs12191243 | 135446826 | C | G | 0.97 | 2691 | 0.23 | 0.35 | 3.81E-12 | -0.23 | 0.03 |
| rs1074849 | 135423412 | G | A | 1.00 | 2691 | 0.23 | 0.55 | 5.11E-12 | -0.22 | 0.03 |
| rs7383186 | 135472502 | A | G | 0.98 | 2691 | 0.17 | 0.78 | 5.79E-12 | 0.25 | 0.04 |
| rs2210366 | 135415208 | G | A | 0.99 | 2691 | 0.25 | 0.50 | 7.59E-12 | -0.22 | 0.03 |
| rs9321485 | 135447773 | T | C | 0.97 | 2691 | 0.23 | 0.48 | 8.41E-12 | -0.22 | 0.03 |
| rs9321486 | 135447820 | T | C | 0.97 | 2691 | 0.23 | 0.45 | 8.53E-12 | -0.22 | 0.03 |
| rs17706858 | 135485094 | C | T | 0.95 | 2691 | 0.17 | 0.68 | 8.78E-12 | 0.25 | 0.04 |
| rs56316290 | 135485498 | G | A | 0.95 | 2691 | 0.17 | 0.68 | 8.84E-12 | 0.25 | 0.04 |
| rs62429816 | 135486022 | A | G | 0.95 | 2691 | 0.17 | 0.63 | 1.01E-11 | 0.25 | 0.04 |
| rs1986846 | 135481885 | T | A | 0.95 | 2691 | 0.17 | 0.78 | 1.03E-11 | 0.25 | 0.04 |
| rs62429811 | 135480054 | T | C | 0.96 | 2691 | 0.17 | 0.78 | 1.15E-11 | 0.25 | 0.04 |
| rs62429812 | 135480147 | T | G | 0.95 | 2691 | 0.17 | 0.78 | 1.17E-11 | 0.25 | 0.04 |
| rs9399140 | 135448643 | T | C | 0.97 | 2691 | 0.23 | 0.48 | 1.18E-11 | -0.22 | 0.03 |
| rs9389272 | 135459837 | G | A | 1.00 | 2691 | 0.17 | 0.79 | 1.34E-11 | 0.24 | 0.04 |
| rs56002646 | 135483809 | T | A | 0.95 | 2691 | 0.17 | 0.73 | 1.44E-11 | 0.25 | 0.04 |
| rs9494149 | 135449315 | C | T | 0.98 | 2691 | 0.23 | 0.48 | 2.02E-11 | -0.22 | 0.03 |
| chr6:135406182:D | 135406182 | TG | T | 0.93 | 2691 | 0.22 | 0.34 | 2.78E-11 | -0.22 | 0.03 |
| rs7383183 | 135472258 | G | A | 0.96 | 2691 | 0.18 | 1.00 | 4.00E-11 | 0.24 | 0.04 |
| rs9376095 | 135450755 | T | C | 0.98 | 2691 | 0.22 | 0.46 | 9.19E-11 | -0.21 | 0.03 |
| chr6:135450891:I | 135450891 | G | GT | 0.97 | 2691 | 0.22 | 0.46 | 1.30E-10 | -0.21 | 0.03 |
| rs55889993 | 135458595 | C | A | 0.99 | 2691 | 0.21 | 0.52 | 1.59E-10 | -0.21 | 0.03 |
| rs1569534 | 135451580 | C | T | 0.99 | 2691 | 0.21 | 0.56 | 1.66E-10 | -0.21 | 0.03 |
| rs1570649 | 135452216 | T | A | 0.99 | 2691 | 0.21 | 0.52 | 2.37E-10 | -0.21 | 0.03 |
| rs6569997 | 135461134 | C | T | 0.99 | 2691 | 0.21 | 0.49 | 3.15E-10 | -0.21 | 0.03 |
| rs9385715 | 135454385 | C | T | 1.00 | 2691 | 0.21 | 0.56 | 3.16E-10 | -0.21 | 0.03 |
| rs9385717 | 135458423 | A | G | 0.99 | 2691 | 0.21 | 0.52 | 3.20E-10 | -0.21 | 0.03 |
| rs9385716 | 135455329 | A | G | 1.00 | 2691 | 0.21 | 0.52 | 3.28E-10 | -0.21 | 0.03 |
| rs7740945 | 135461866 | C | A | 0.99 | 2691 | 0.21 | 0.49 | 3.29E-10 | -0.21 | 0.03 |
| rs1883355 | 135461937 | A | G | 0.99 | 2691 | 0.21 | 0.49 | 3.30E-10 | -0.21 | 0.03 |
| rs6909822 | 135463333 | G | A | 0.99 | 2691 | 0.21 | 0.49 | 3.35E-10 | -0.21 | 0.03 |
| rs2078213 | 135458071 | T | C | 1.00 | 2691 | 0.21 | 0.52 | 3.75E-10 | -0.21 | 0.03 |
| rs2050019 | 135463947 | C | T | 0.99 | 2691 | 0.21 | 0.49 | 3.76E-10 | -0.21 | 0.03 |
| rs6929404 | 135454027 | C | A | 1.00 | 2691 | 0.21 | 0.48 | 3.78E-10 | -0.21 | 0.03 |
| rs9373125 | 135452892 | T | C | 0.99 | 2691 | 0.21 | 0.56 | 3.91E-10 | -0.21 | 0.03 |
| rs6914717 | 135456758 | A | G | 1.00 | 2691 | 0.21 | 0.48 | 4.07E-10 | -0.21 | 0.03 |
| rs2026310 | 135465551 | A | C | 0.99 | 2691 | 0.21 | 0.53 | 4.07E-10 | -0.21 | 0.03 |
| rs9376096 | 135469752 | C | T | 0.99 | 2691 | 0.21 | 0.53 | 4.36E-10 | -0.21 | 0.03 |
| rs7748169 | 135471616 | T | C | 0.99 | 2691 | 0.21 | 0.56 | 5.38E-10 | -0.21 | 0.03 |
| rs139756487 | 135474634 | T | C | 0.98 | 2691 | 0.21 | 0.53 | 5.61E-10 | -0.21 | 0.03 |
| chr6:135467218:I | 135467218 | T | TGA | 0.97 | 2691 | 0.21 | 0.45 | 5.90E-10 | -0.21 | 0.03 |
| rs9373128 | 135476440 | A | T | 0.97 | 2691 | 0.21 | 0.53 | 6.22E-10 | -0.21 | 0.03 |
| chr6:135476379:I | 135476379 | C | CT | 0.97 | 2691 | 0.21 | 0.53 | 6.33E-10 | -0.21 | 0.03 |
| rs9389274 | 135477207 | G | A | 0.97 | 2691 | 0.21 | 0.52 | 8.75E-10 | -0.21 | 0.03 |
| rs41294856 | 135412382 | C | T | 0.91 | 2691 | 0.10 | 0.74 | 1.28E-09 | -0.30 | 0.05 |
| rs41294858 | 135412636 | T | C | 0.91 | 2691 | 0.10 | 0.74 | 1.33E-09 | -0.30 | 0.05 |
| rs41294860 | 135414069 | C | G | 0.91 | 2691 | 0.10 | 0.66 | 1.35E-09 | -0.30 | 0.05 |
| rs41294854 | 135401371 | C | T | 0.90 | 2691 | 0.10 | 0.74 | 1.36E-09 | -0.30 | 0.05 |
| rs56076748 | 135414209 | G | A | 0.91 | 2691 | 0.10 | 0.66 | 1.36E-09 | -0.30 | 0.05 |
| rs76732255 | 135408472 | G | T | 0.91 | 2691 | 0.10 | 0.74 | 1.40E-09 | -0.30 | 0.05 |
| rs75375208 | 135403089 | C | G | 0.90 | 2691 | 0.10 | 0.74 | 1.40E-09 | -0.30 | 0.05 |
| rs113033196 | 135403927 | T | C | 0.90 | 2691 | 0.10 | 0.74 | 1.41E-09 | -0.30 | 0.05 |
| rs79562575 | 135404112 | G | A | 0.90 | 2691 | 0.10 | 0.74 | 1.41E-09 | -0.30 | 0.05 |
| rs76267242 | 135417460 | G | T | 0.91 | 2691 | 0.10 | 0.66 | 1.98E-09 | -0.29 | 0.05 |
| rs55731938 | 135414850 | G | A | 0.90 | 2691 | 0.11 | 1.00 | 7.34E-09 | -0.27 | 0.05 |
| rs1547247 | 135390836 | G | A | 0.94 | 2691 | 0.31 | 0.42 | 2.31E-08 | 0.17 | 0.03 |
