# Supplementary material for: Erythropoietin in the General Population: Reference Ranges and Clinical, Biochemical and Genetic Correlates
Source: PLoS One. 2015 Apr 27;10(4):e0125215. doi: 10.1371/journal.pone.0125215 (PMC4411129; doi:10.1371/journal.pone.0125215)
Supplement: S7 Table — (DOCX) [file pone.0125215.s007.docx]

| **Supplemental Data Table 7: Significant Trans eQTL’s (FDR < 0.05) for rs7776054 detected in non-transformed peripheral blood samples from 5,311 individuals.** | | | | | | | | |
| --- | --- | --- | --- | --- | --- | --- | --- | --- |
| **Probe Chr.** | **Probe Chr. position** | **SNP Alleles** | **Minor Allele** | **Z-score** | **Gene name** | **P-value** | **FDR** | **Function/connection to erytropoesis** |
| 6 | 37250791 | A/G | G | -10.28 | PIM1 | 8.27E-25 | 0 | PIM1 knock-out mice exhibit subtle erythrocyte microcytosis and appears to be a mediator of cytokine-induced cell growth. [1-3] |
| 19 | 2376105 | A/G | G | -9.49 | TMPRSS9 | 2.31E-21 | 0 |  |
| 22 | 23266606 | A/G | G | -8.82 | C22orf13 | 1.1E-18 | 0 |  |
| 18 | 32630599 | A/G | G | 8.56 | C18orf10 | 1.15E-17 | 0 |  |
| 1 | 47422173 | A/G | G | -8.55 | PDZK1IP1 | 1.24E-17 | 0 | Also known as Map17 which has a role in erythropoiesis in zebrafish as knockdown caused a reduction in the number of circulating erythrocytes. Knockout of a MAP17 enhancer (Scl^Δ40/Δ40^) in mice resulted in reduced numbers of erythroid CFU and a slight increased EPO levels; the embryonic stem cells revealed impaired erythroid differentiation. PDZK1IP1 protein shares transcriptional enhancer elements with the blood stem cell regulator TAL1/SCL. [4,5] |
| 1 | 246106142 | A/G | G | -8.43 | TRIM58 | 3.35E-17 | 0 | Identified in a GWAS on red blood cell traits for RBC [6] |
| 2 | 219859771 | A/G | G | -7.43 | DNAJB2 | 1.1E-13 | 0 |  |
| 20 | 55417256 | A/G | G | -7.18 | AL109955.37-3,RBM38 | 7.23E-13 | 0 | Identified in a GWAS on red blood cell traits for MCV [6] |
| 20 | 55417488 | A/G | G | -7.09 | AL109955.37-3,RBM38 | 1.31E-12 | 0 | (see probe 55417256) |
| 1 | 21795363 | A/G | G | 7.03 | RAP1GAP | 2.03E-12 | 0 | RAP1GAP negatively controls RAP1 which provides many roles in the haematological processes such as such as cell proliferation, adhesion, differentiation, and embryogenesis. [7,8] |
| 11 | 63844774 | A/G | G | -6.91 | PRDX5 | 4.85E-12 | 0 |  |
| 6 | 30227895 | A/G | G | -6.61 | TRIM10 | 3.83E-11 | 0 | TRIM10 plays a role in differentiation of erythroid cells, [9] |
| 11 | 5219917 | A/G | G | 6.37 | - | 1.85E-10 | 0 |  |
| 22 | 31224455 | A/G | G | -6.09 | FBXO7 | 1.11E-09 | 0 | Identified in a GWAS on red blood cell traits for MCH [6] |
| 11 | 63845367 | A/G | G | -6.04 | PRDX5 | 1.55E-09 | 0 |  |
| 7 | 16789879 | A/G | G | -5.92 | TSPAN13 | 3.25E-09 | 0 |  |
| 16 | 55200045 | A/G | G | -5.89 | MT2A | 3.9E-09 | 0 |  |
| 1 | 159407830 | A/G | G | -5.86 | B4GALT3 | 4.5E-09 | 0 |  |
| 8 | 21995803 | A/G | G | -5.7 | EPB49 | 1.22E-08 | 0 |  |
| 8 | 86428105 | A/G | G | 5.7 | CA1 | 1.22E-08 | 0 | CA1 is highly expressed in erythrocytes and known to interact with hbA, |
| 19 | 4396589 | A/G | G | -5.56 | UBXN6 | 2.73E-08 | 0 | Identified in a GWAS on red blood cell traits for MCV.[6] |
| 1 | 6443519 | A/G | G | -5.55 | ESPN | 2.83E-08 | 0 |  |
| 23 | 37475852 | A/G | G | 5.51 | XK | 3.49E-08 | 0.01 | Reported to control synthesis of the Kell blood group |
| 7 | 100669809 | A/G | G | -5.5 | FIS1 | 3.76E-08 | 0.01 |  |
| 17 | 44656026 | A/G | G | 5.44 | PHOSPHO1 | 5.27E-08 | 0.01 |  |
| 11 | 63845375 | A/G | G | -5.42 | PRDX5 | 5.97E-08 | 0.01 |  |
| 16 | 4787220 | A/G | G | -5.34 | ROGDI | 9.07E-08 | 0.01 |  |
| 11 | 60782494 | A/G | G | -5.28 | VWCE | 1.28E-07 | 0.02 |  |
| 7 | 142348516 | A/G | G | 5.22 | KEL | 1.81E-07 | 0.02 | Resposible for the Kell blood group |
| 20 | 56919270 | A/G | G | 5.11 | GNAS,GNAS | 3.27E-07 | 0.03 |  |

**References**

1. Magnuson NS, Wang Z, Ding G, Reeves R. (2010) Why target PIM1 for cancer diagnosis and treatment? Future Oncol 6: 1461-1478.

2. An N, Lin YW, Mahajan S, Kellner JN, Wang Y, et al. (2013) Pim1 serine/threonine kinase regulates the number and functions of murine hematopoietic stem cells. Stem Cells 31: 1202-1212.

3. Sathyanarayana P, Dev A, Fang J, Houde E, Bogacheva O, et al. (2008) EPO receptor circuits for primary erythroblast survival. Blood 111: 5390-5399.

4. Tijssen MR, Cvejic A, Joshi A, Hannah RL, Ferreira R, et al. (2011) Genome-wide analysis of simultaneous GATA1/2, RUNX1, FLI1, and SCL binding in megakaryocytes identifies hematopoietic regulators. Dev Cell 20: 597-609.

5. Ferreira R, Spensberger D, Silber Y, Dimond A, Li J, et al. (2013) Impaired in vitro erythropoiesis following deletion of the scl (Tal1) +40 enhancer is largely compensated for in vivo despite a significant reduction in expression. Mol Cell Biol 33: 1254-1266.

6. van der Harst P, Zhang W, Mateo Leach I, Rendon A, Verweij N, et al. (2012) Seventy-five genetic loci influencing the human red blood cell. Nature 492: 369-375.

7. Shao X, Miao M, Qi X, Chen Z. (2012) Ras-proximate-1 GTPase-activating protein and Rac2 may play pivotal roles in the initial development of myelodysplastic syndrome. Oncol Lett 4: 289-298.

8. Stork PJ, Dillon TJ. (2005) Multiple roles of Rap1 in hematopoietic cells: Complementary versus antagonistic functions. Blood 106: 2952-2961.

9. Blaybel R, Theoleyre O, Douablin A, Baklouti F. (2008) Downregulation of the spi-1/PU.1 oncogene induces the expression of TRIM10/HERF1, a key factor required for terminal erythroid cell differentiation and survival. Cell Res 18: 834-845.
