# Supplementary figures and images for: Erythropoietin in the General Population: Reference Ranges and Clinical, Biochemical and Genetic Correlates
Source: PLoS One. 2015 Apr 27;10(4):e0125215. doi: 10.1371/journal.pone.0125215 (PMC4411129; doi:10.1371/journal.pone.0125215)

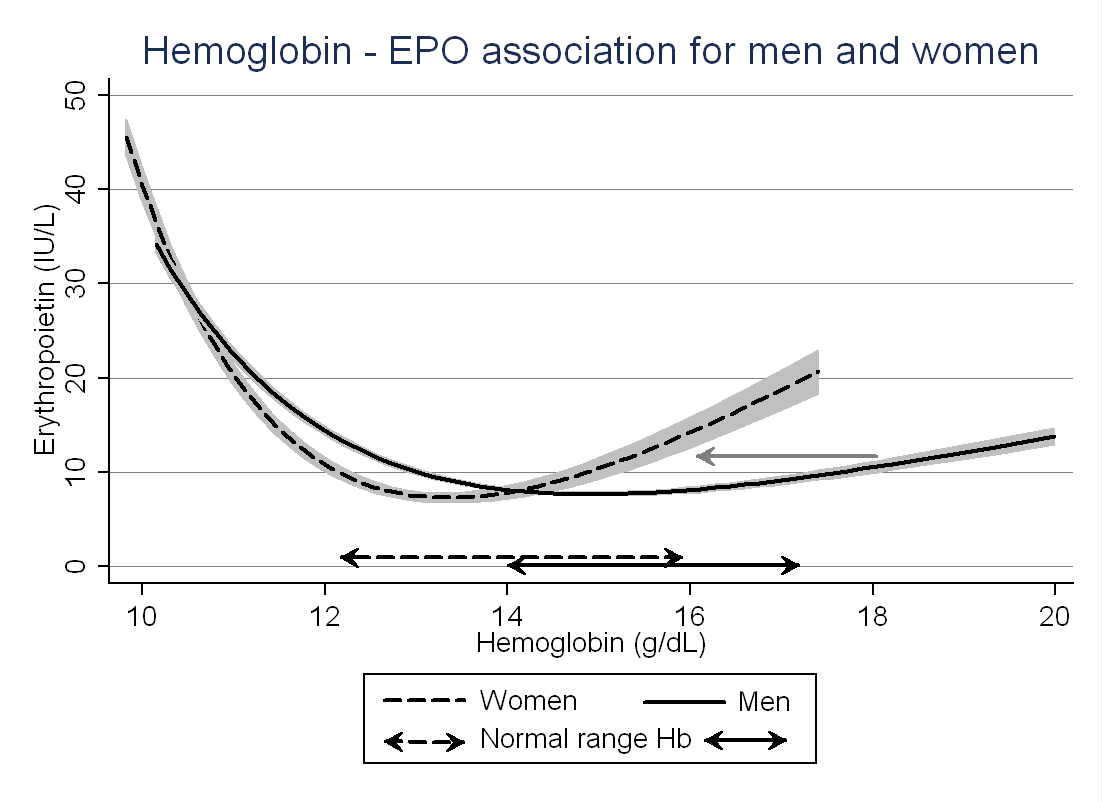

Supplement: S1 Fig — The association between hemolgobin and erythropoietin separate for men (solid line) and women (dashed line); the shift of the association is depicted by the gray arrow. The normal range of hemoglobin levels is displayed by the black lines with both sided arrow heads. (TIF) [file pone.0125215.s008.tif]
